# Genome-scale examination of NBS-encoding genes in blueberry

**DOI:** 10.1038/s41598-018-21738-7

**Published:** 2018-02-21

**Authors:** Jose V. Die, Belén Román, Xinpeng Qi, Lisa J. Rowland

**Affiliations:** 1 Genetic Improvement Fruits and Vegetables Lab. U.S. Department of Agriculture, Agricultural Research Service, Beltsville, MD USA; 2 Crop Breeding and Biotechnology Department, IFAPA Research Centre Alameda del Obispo, Córdoba, Spain

## Abstract

Blueberry is an important crop worldwide. It is, however, susceptible to a variety of diseases, which can lead to losses in yield and fruit quality. Although screening studies have identified resistant germplasm for some important diseases, still little is known about the molecular basis underlying that resistance. The most predominant type of resistance (*R*) genes contains nucleotide binding site and leucine rich repeat (NBS-LRR) domains. The identification and characterization of such a gene family in blueberry would enhance the foundation of knowledge needed for its genetic improvement. In this study, we searched for and found a total of 106 NBS-encoding genes (including 97 NBS-LRR) in the current blueberry genome. The NBS genes were grouped into eleven distinct classes based on their domain architecture. More than 22% of the NBS genes are present in clusters. Ten genes were mapped onto seven linkage groups. Phylogenetic analysis grouped these genes into two major clusters based on their structural variation, the first cluster having toll and interleukin-1 like receptor (TIR) domains and most of the second cluster containing a coiled-coil domain. Our study provides new insight into the NBS gene family in blueberry and is an important resource for the identification of functional *R*-genes.

## Introduction

Blueberries (*Vaccinium* spp.) have become a major crop worldwide. Demand for blueberries in the last decades has exceeded that predicted from prior growth. Increased consumption has been driven largely by a greater public awareness of health benefits associated with eating blueberries. This expansion has been evident worldwide (increase of 58.0% from 2009–2014) and in the U.S. (increase of 32.5% from 2010–2015). In 2016, U.S. production reached 296,000 metric tons with a market value of $720 million^1^.

The expansion of blueberry production around the world raises concerns regarding distribution of existing diseases as well as the emergence of new ones^2^. Highbush (*V*. *corymbosum* L.), lowbush (*V*. *angustifolium* Ait.) and rabbiteye (V. *virgatum* Ait., syn. *V*. *ashei* Reade) blueberries are susceptible to attack by an array of fungal, bacterial, and viral diseases some specific to blueberries, while others are general within the genus *Vaccinium*^3^. At present, sanitation and other cultural practices are the primary means of disease management available to blueberry propagators and growers^4,5^. However, these practices alone are often insufficient to prevent disease outbreaks and their associated losses in terms of yield and fruit quality.

Currently, it is generally accepted that genetic resistance is the most effective means to control diseases. Plant disease resistance can be addressed through multiple approaches. A durable host resistance can be achieved by plant breeders, pathologists and geneticists working together with the goal of pyramiding multiple resistance genes (*R*) within commercial cultivars. There is a growing perception that a multiple gene-for-gene model is more appropriate for developing strategies to promote durable resistance in agriculture^6^. Pyramiding should be especially effective when particular *R* genes are able to recognize multiple avirulence effectors and/or multiple pathogen isolates^7–9^.

For some important diseases affecting blueberry, resistant germplasm has been identified and characterized through screening studies^5,10,11^. However, very little is known about the molecular basis underlying that resistance and no detailed analysis of *R*-gene characteristics is currently available for the improvement of blueberry.

The NBS-LRR genes, which contain a nucleotide-binding site (NBS) and leucine–rich repeats (LRRs), are the largest class of *R*-genes. The deduced NBS-LRR proteins can be divided into two subfamilies, TIR-NBS-LRR (TNL) and non-TNL-NBS-LRR (nTNL), based on their N-terminal features^12^. TNLs possess a domain with similarity to both the intracellular signaling domains of *Drosophila* Toll and the mammalian Interleukin-1 receptor (TIR), whereas nTNLs often have a putative N-terminal coil-coil (CC) domain and are thus designated CNLs. The LRR motif is typically involved in protein-protein interactions and responsible for recognition specificity^13^. It is generally accepted that the primary function of the highly conserved NBS domain is to control the signal transduction through conformational change^14–18^. Recent studies suggest that the NBS region also might play a role in the establishment of resistance specificity^19–22^.

This study provides insight into the NBS-LRR gene family in blueberry. We used an iterative process of manual and computational analysis to identify members of the blueberry NBS-LRR-encoding gene family within the recently released blueberry draft genome sequence. A phylogenetic tree was constructed and the NBS-LRR-encoding genes were separated into two distinct groups. Conserved motifs, structural diversity and protein domain architectures were further analyzed in both families to support the partition. The findings will provide a strong groundwork for the isolation of candidate *R*-genes in blueberry.

## Results

### Total number of NBS-encoding genes and architectural diversity in blueberry

One hundred and six NBS-encoding genes, including 97 NBS-LRR sequences were identified in the blueberry genome. Among the 97 NBS-LRR, 11 were identified as TNL resistance-like genes, including TNL and TCNL, and 86 nTNL, including CNL, CNCL, XNL, NLNL, NL and RPW8 genes. In addition 9 NBS type genes, which lacked the LRR domain, were detected containing TCN, CN and XN (Table 1). Approximately 25,000 protein-encoding genes are estimated in the draft genome assembly^23^. Therefore, the NBS-LRR genes accounted for approximately 0.4% of the predicted ORFs. The average number of exons detected in the NBS-LRR genes was 2.01. Because the genome assembly is still a work in progress, it is difficult to know how this estimate compares to that in all predicted blueberry genes. The average TNL exon number (3.73) was greater than that in nTNLs (1.75; *t* test, *P* < 0.0005). The pattern of TNL exons outnumbering CNL exons has also been observed in other woody species, such as grape and poplar^24^, or apple, pear and peach^25^.

**Table 1:** Number and classification of NBS-encoding genes in the blueberry genome.

Predicted protein domain	Letter code	N. of genes
**NBS-LRR type genes**		
TIR-NBS-LRR subclass		
TIR-NBS-LRR	TNL	10
TIR-CC-NBS-LRR	TCNL	1
non-TIR-NBS-LRR subclass		
CC-NBS-LRR	CNL	53
X-NBS_CC_-LRR	XNL	20
NBS_CC_-LRR	NL	10
CC-NBS-CC-LRR	CNCL	1
NBS-LRR-NBS-LRR	NLNL	1
RPW8-NBS-LRR	RNL	1
**NBS type genes**		
CC-NBS	CN	5
TIR-CC-NBS	TCN	2
X-NBS	XN	2
**Total NBS genes**		**106**

TIR Toll/interleukin-1-receptor, CC coiled-coil domain, LRR leucine-rich repeat domain, NBS nucleotide-binding site. CN, TCN and XN are structurally incomplete genes. NL and XNL are sequences with the N-terminal region comparable in length to intact CNL but we could not identify the CC domain based on the COILS program. The CC domain was detected in XNL sequences only by the Conserved Domain database at NCBI.

**Figure 1:**
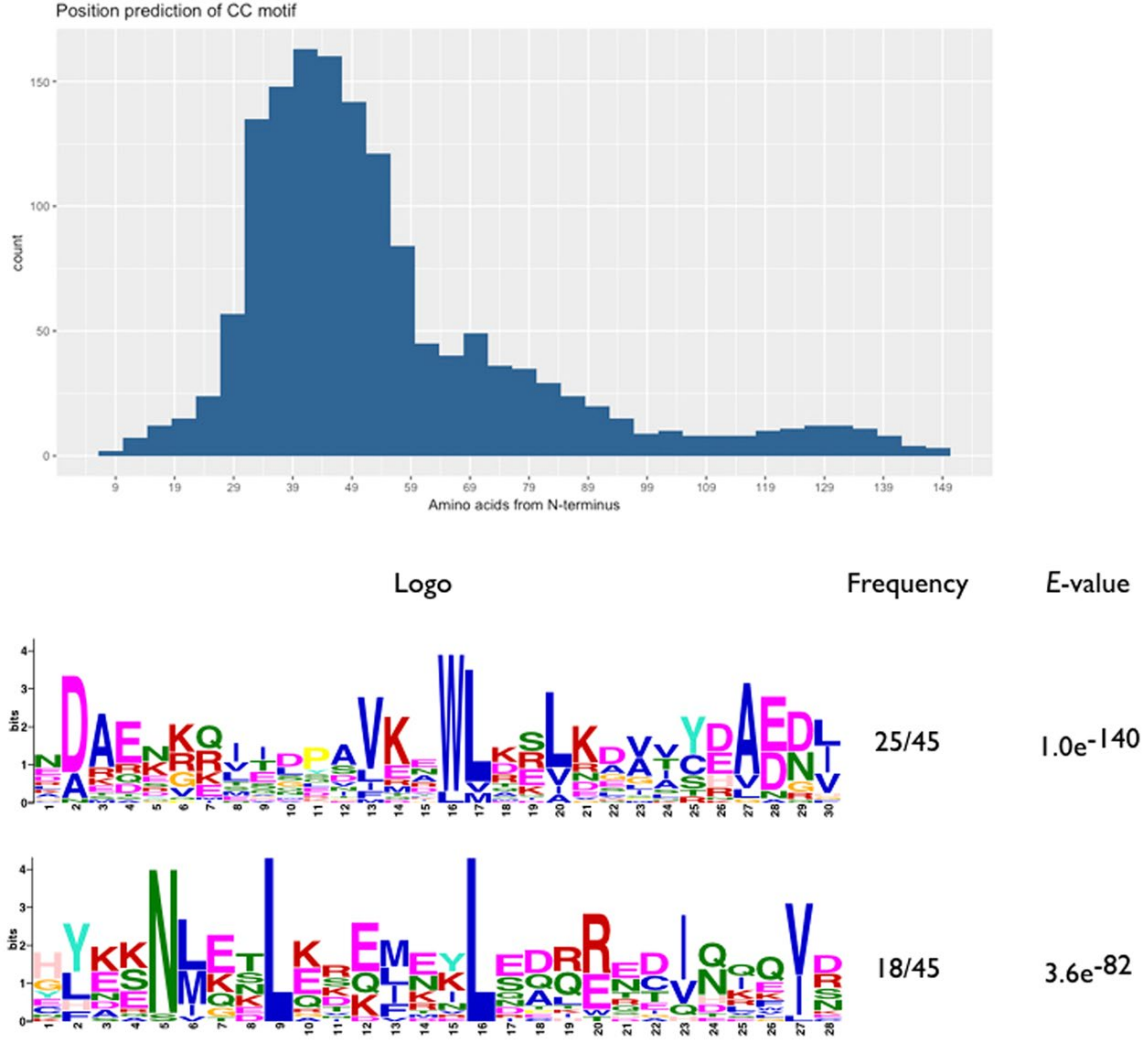
Analysis of the N-terminal domain in non-TNL sequences. (**a**) Amino acid position of predicted CC motif. (**b**) Regular expression of the 30–60 amino acids region from blueberry CNL sequences. Sequence logo representation was generated from multiple alignments with MEME software.

The presence of a CC domain has been identified as a characteristic motif in the N terminus of the nTNL proteins. We found strong evidence for a CC domain in 54 out of the 86 non-TNL proteins in blueberry. The majority of these 54 proteins show the predicted CC motif positioned from ~30–60 amino acids from the N terminus of the protein. Using the MEME suite for motif-based analysis, we identified one distinct motif spanning mostly positions 30–60 [DA]AE[NK][KRG]-x(5)-VK-x-WL-x(2)-LK[DA][VA][VTA][YCS][DEH][AV][ED][DNG][LIV], which was coincident with the CC pattern identified by COILS software. A second common motif was also present in some of the CNLs in roughly in the same positions (30–60), suggesting that different expressions may confer the CC domain (Fig. 1). Another interesting feature from the MEME analysis concerned the last residue of the kinase-2 in the NBS domain. That residue has been used to predict whether a given protein belongs to the TNL (D, aspartate) or CNL (W, tryptophan) subfamilies^26,27^. The results of this study are consistent with those reports. The ‘D’ residue was found in all the TNL proteins showing the kinase-2 motif, whereas the ‘W’ residue was the predominant one in the CNL proteins (Supplementary Fig. S1).

### NBS blueberry genes and comparison with other species

**Figure 2:**
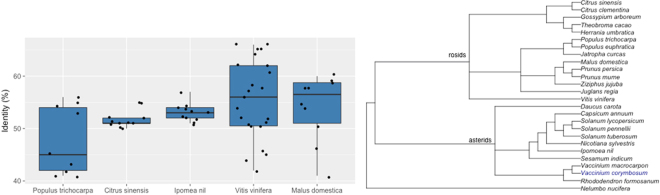
Comparative analysis between blueberry NBS sequences and other species. (**a**) Boxplot of identity distribution scores by plant RefSeq database. Blueberry NBS sequences were used as queries against the RefSeq database and the identity from the best-matching NBS protein for each blueberry sequence was recorded. Figure shows the five species with higher number of hits. (**b)** Species tree of some species in which homologs of NBS-LRR genes have been identified. The data were downloaded from NCBI Common Tree in the Taxonomy section (http://www.ncbi.nlm.nih.gov/taxonomy) and the tree was constructed using the R package “ape”^30^.

Since all plant *R*-like genes are assumed to have originated from one common ancestor^28^, NBS-LRR genes provide a good opportunity to explore relationships between blueberry and other plant genomes. For this, we searched the blueberry proteins against the well-annotated plant reference sequence protein RefSeq database^29^ and identified the best scoring protein for each. Figure 2a shows the distribution of identity scores obtained for the best-matching sequences from the 5 species that accumulated the most hits. Apple proteins had the highest median percent identity scores, followed by grape, morning glory, sweet orange and poplar. Grape proteins had the highest number of hits with blueberry proteins (23 hits). From the asterids clade, the highest number of RefSeq hits were for morning glory (11 hits) and carrot (4 hits), whereas other species expected to be more closely related to blueberry, such as cranberry or rhododendron, in the same Ericaceae family, did not show any hit (Fig. 2b). Results from searching against the Genbank nr protein database are shown in Supplementary Fig. S2.

### Distribution of NBS-encoding genes across linkage groups

**Figure 3:**
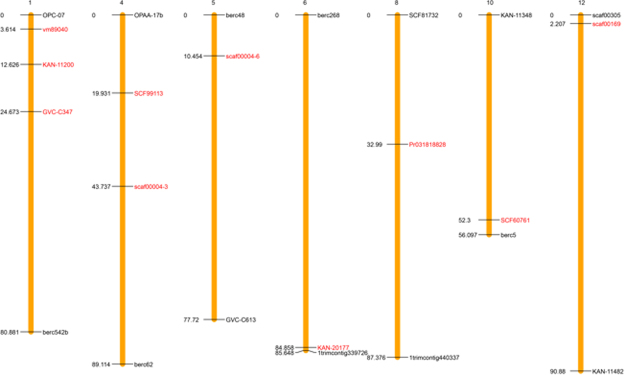
Position of 10 NBS-LRR genes on the blueberry linkage groups. Linkage map of F1#10 × W85-23 diploid population. First and last marker on each linkage group (cM) are shown as references. Positions of NBS genes are shown in red color. Only linkage groups with NBS markers are shown.

At this time, the blueberry genome has not been assembled into chromosomes or pseudo molecules but into small scaffolds. This is a constraint to studying the distribution of NBS among chromosomes because most genes reside on different scaffolds. However, by mapping all identified NBS genes onto the assembled scaffolds, we observed a trend consisting of NBS gene clusters. In blueberry more than 22% of the NBS genes appear together (on the same scaffold) with at least one other NBS gene. The larger clusters were found on four scaffolds (containing 3 NBS sequences each, ~11% NBS identified in this study), while six other scaffolds contained 2 NBS genes each. The rest of the NBS genes are tentatively organized as singletons. To anchor scaffolds onto the most recent diploid genetic linkage map, we used an approach based on *in silico* PCR. Two hundred SSR markers (out of 1093 primer pairs from 13 linkage groups or LGs) were located in only one position in 174 scaffolds, representing ~79.9 Mb in genomic length. From that group, we found 10 SSR markers on the same scaffolds as 10 NBS-encoding genes. From the position of the markers, we placed 1 NBS gene on each of LGs 5, 6, 8, 10 and 12, while LGs 4 and 1 had 2 and 3 NBS genes, respectively (Fig. 3).

### NBS gene duplication

To identify duplicated gene pairs, we defined a gene family according to the criteria shown in the Methods section. Eighteen NBS gene families were identified including 56 NBS genes. The results revealed that the percentage of NBS genes in multi-gene families (56/106 = 52.8%) was significantly lower than those in poplar (78.1%) or grape (77.8%), higher than those in *Arabidopsis* (47.3%) or *Prunus* genomes (48.3%), and in the same range as those reported in chestnut (52.6%^25^). For the multi-gene families, the maximum number of family members was six and the average number of family members was 3.11, which (with the exception of *Arabidopsis*) is slightly lower than the average number of family members in the other species. This suggests that the multi-gene blueberry families are comprised of fewer members than those of the other woody species (Table 2).

**Table 2:** Organization in families of NBS-encoding genes in five plant genomes.

Organization	Blueberry		*Arabidopsis* ^a^		Grapevine^a^		Poplar^a^		Chestnut^b^		*P*. *mume*^c^	
	70%	80%	70%	80%	70%	80%	70%	80%	70%	80%	70%	80%
Single-genes	50	68	93	134	119	172	91	106	246	304	182	241
Multi-genes	56	38	81	40	416	363	325	310	273	215	170	111
Gene families	18	14	25	15	94	102	61	64	64	61	49	39
Max. family members	6	4	7	4	13	10	23	17	19	17	8	7
Avg. family members	3.11	2.71	3.24	2.67	4.43	3.56	5.33	4.84	4.27	3.52	3.47	2.85
Multi-genes/single genes	1.12	0.56	0.87	0.30	3.50	2.11	3.57	2.92	1.11	0.71	0.93	0.46
% Multi-gene families	52.8	35.8	46.6	23.0	77.8	67.9	78.1	74.5	52.6	41.4	48.3	31.5
% TNL multi-genes	18.2	0							14.8	7.4	53.6	29.4
% non-TNL multi-genes	62.8	44.2							54.7	43.3	44.2	33.1

^a^Data from^24^.

^b^Data from^61^.

^c^Data from^25^.

To distinguish more recent duplications, we used a more stringent criterion for defining a multi-gene family (coverage and identity of 80% compared to 70%). In this case, the proportion of multi-genes among all NBS-encoding genes decreased in all the species (decrease ranging from ~5% to 51%). However, we could still place 35.8% of blueberry genes into multi-gene families with this strict criteria (Table 2). Moreover, the number of both multi-gene families and multi-genes in the non-TNL group was greater than those in the TNL group, indicating that duplication of NBS-encoding genes occurred essentially among non-TNL genes.

It is noteworthy that the maximum number of members in a family across similarities/coverage thresholds is smaller in blueberry than in the other woody species, which are shown in Table 2. However, when we use the stringent criteria (80% compared to 70%), blueberry is one of the species (along with poplar) showing a very small decrease in the average number of genes in a family. This seems to indicate that the expansion of NBS genes through gene duplication is mainly caused by an increase in groups and not by an increase in the number of genes within a group.

### Phylogenetic analysis

**Figure 4:**
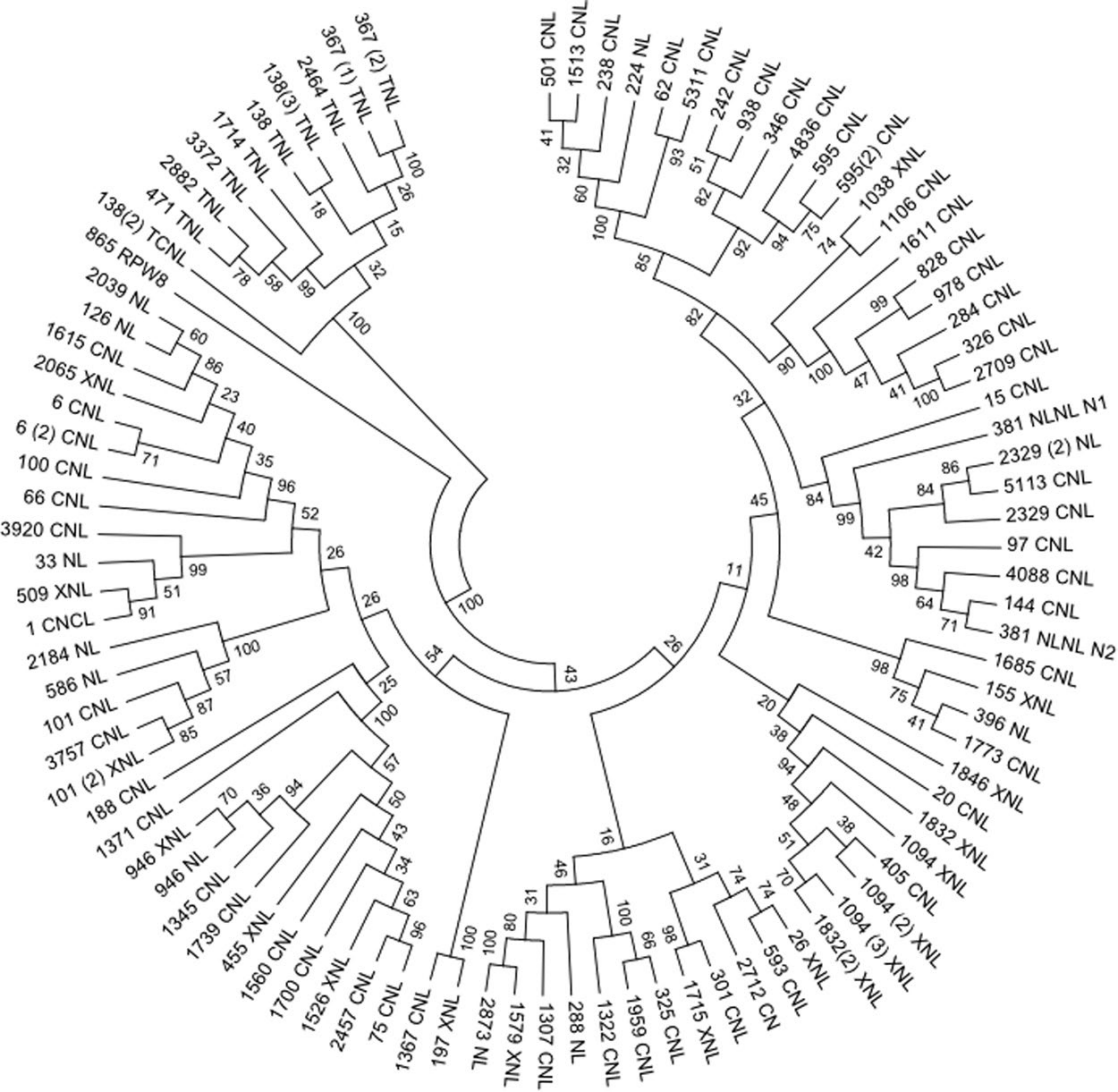
Phylogenetic tree of NBS genes in the blueberry genome. The tree is based on the maximum likelihood method using MEGA software. Numbers on the branches indicate the percentage of 1000 bootstrap replicates. Gene names are intended to represent blueberry scaffolds and domain configurations. Numbers between brackets denote more than one gene per scaffold.

A phylogenetic tree using the conserved NBS domains was constructed to examine the evolutionary relationships among the NBS genes. After removing short sequences with deletions, 94 NBS sequences were finally used. Two clades were distinctly separated (bootstrap = 100%) in the phylogenetic reconstruction (Fig. 4). The TNL subclass included 9 TNLs and 1 TCNL. Within the nTNL clade, two groups were separated, suggesting two different ancient nTNL ancestors in blueberry. All XNLs or NLs were clustered in the nTNL, indicating that those genes might be nTNL-related. The two nTNL groups were further organized into two subclusters. The only RNL blueberry sequence (865RPW8) classified into the nTNL subclass is not embedded into a CNL subcluster, but formed a separated lineage within a CNL subgroup on the tree. All members of each multi-gene-family clustered together in the tree. To infer the time elapsed since gene duplication and detect the mode of selection, we estimated the Ks values for each pair of duplicates within a gene family. The Ks values peaked at 0.25–0.35 time frames (>52% of duplicates; Supplementary Fig. S3).

### Blast2GO annotation

We annotated the blueberry NBS proteins using the annotation platform Blast2GO to gain further insights into their potential biological meaning. The analysis assigned GO terms to the whole set of sequences, so the 106 proteins could be classified into one or more ontologies (Biological Process, Molecular Function, and Cellular Component). Plots summarizing GO categories are shown in Supplementary Fig. S4. Concerning the Biological Process, “defense response”, “signal transduction” and “cell communication” were the GO terms most often assigned to the sequences. As for Molecular Function, the whole 106 proteins were assigned to five different binding-related GO terms, which emphasizes the highly variable binding nature of these proteins. Finally, regarding the Cellular Component, only 4 sequences were classified under this ontology. They were associated with “membrane”.

Next, we aimed to explore whether our dataset contained significantly enriched gene ontologies. For this, we compared our annotation to some well-annotated blueberry transcriptome datasets including ~1,500 sequences from flower buds exposed to 0 and 400 chill units, respectively^31^ and >18,000 transcripts from fruits regulated during berry development^32^. Using a FDR cutoff of <0.01, the NBS genes contained an overabundance of the terms ‘ADP binding’, ‘defense response’ and ‘signal transduction’ (Supplementary Table S1).

### Analysis of regulatory elements in the promoter regions

We screened the proximal and distal regions of promoters (up to 1500 bp upstream of the transcriptional initiation site, TSS) to identify candidate *cis*-regulatory elements (CRE) that might contribute to the fine regulation of gene expression at the transcriptional level. We were able to retrieve the promoter genomic sequences from 65 NBS-encoding genes, including 5 NBS-type, 5 TNLs, and 55 nTNLs. Custom Python scripts were used to parse the output fasta file containing the promoter regions. We estimated the number, abundance and position of CREs that had been associated previously with plant defense mechanisms in the literature. Further statistical analysis indicated an enriched content of 9 CREs in the NBS gene promoters of blueberry (Table 3). All the promoters contained CACTFTPPCA1 and DOFCOREZM, which appeared a total of 727 and 621 times, respectively in the whole set of 65 promoters. On the other hand, some regulatory elements were present in only a few of the sequences such as ABRE (n = 6), and ATHB5 (n = 4). However, these two CREs were also clearly overrepresented in our query set.

**Table 3:** Conserved *cis*-regulatory elements (CRE) in blueberry NBS-encoding gene promoters.

CRE	Motif	Query^1^	Promoters Observed	Total Occurrences Observed^2^	Avg. Occurrs. per Promoter	Total Occurrences Expected^3^	Enrichment Factor^4^	*P*-value^5^
GT1GMSCAM4	GAAAAA	65	48	102	2.13	50.64	2.01	0.011
ABRE	ACGTGTC	65	6	8	1.33	4.10	1.95	0.009
MYCATERD1	CATGTG	65	26	31	1.19	18.69	1.66	0.010
Motif CTCTT	CTCTT	65	58	172	2.97	117.36	1.47	0.014
CAATBOX 1	CCAAT	65	56	133	2.38	107.22	1.24	0.015
G-box	CACATG	65	21	24	1.14	20.05	1.20	0.012
DOFCOREZM	AAAG	65	65	621	9.55	520.10	1.19	0.015
ATHB5	CAATNATTG	65	4	4	1.00	3.41	1.17	0.013
RAV1AAT	CAACA	65	54	111	2.06	104.03	1.07	0.015
CACTFTPPCA1	YACT	65	65	727	11.18	930.09	0.78	—

^1^Total number of promoters in the query set.

^2^Total number of motifs in the query set.

^3^Total number of motifs expected to occur by chance/1.5 kb promoter based on nucleotide frequency in 65 blueberry promoter sequences.

^4^Number of motifs observed divided by the number of motifs expected to occur by chance.

^5^Probabilities based on 2,000 Monte Carlo simulations.

**Figure 5:**
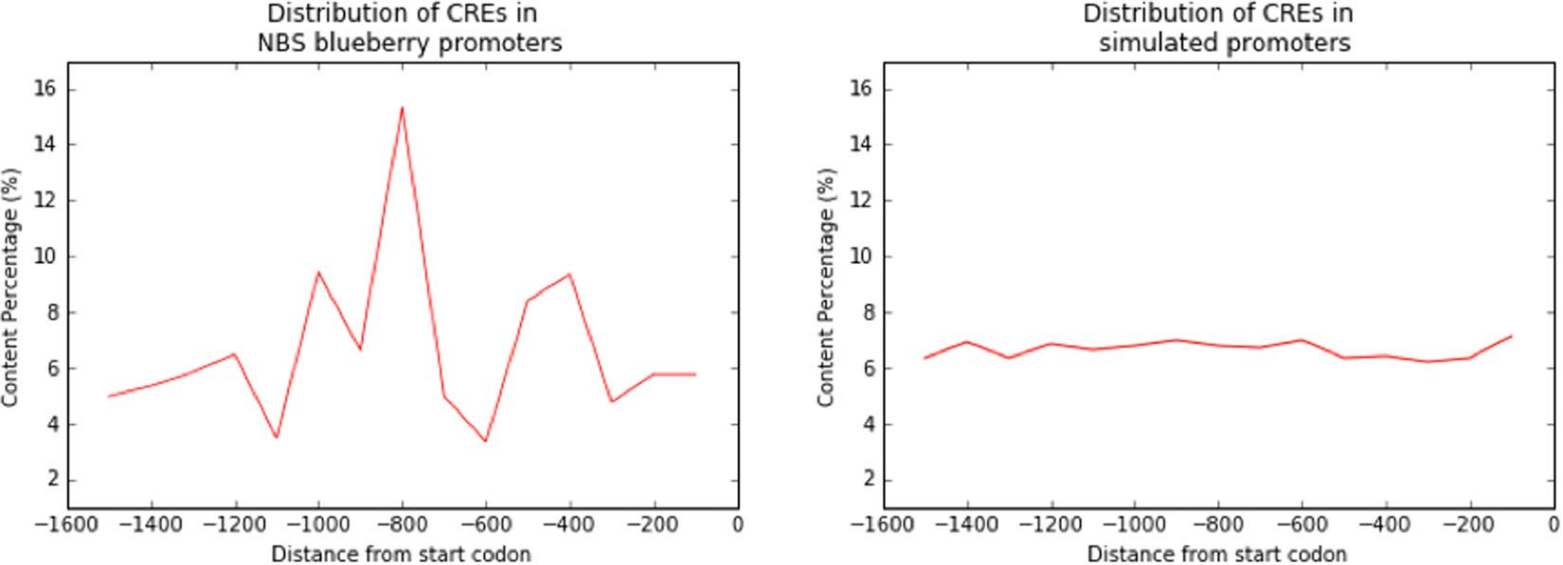
Distribution of CREs in the blueberry promoters data set and simulated control set. The control dataset is based on 2000 simulations, where each simulation contains n = 65 sequences, with length = 1500 bp per sequence, and an expected frequency GC = 0.37.

We then tested whether any given CRE is more common in certain promoter regions compared to background. For this, the promoter sequences were divided into 100 bp nucleotide fragments and the content of the CREs enriched in our dataset was calculated. An increase in density of the fragments was identified at a distance of ~−900 to −800 from the TSS (Fig. 5).

## Discussion

So far, there have been no disease resistance QTL identified by association or recombinant mapping in blueberry. A genome-scale search for blueberry *R*-like genes provides a useful database of potential resistance genes and candidate molecular markers for further testing in the development of marker-assisted strategies for selection of disease resistance. The data mining approach using available draft genome versions is a common strategy to predict NBS gene models. The approach has been used in pigeonpea^33^, sweet orange^34^, kiwifruit^35^ and *Eucalyptus grandis*^36^, to name a few recent examples.

This work is a comprehensive genome-scale examination of NBS-encoding genes in blueberry. We identified 106 NBS-encoding genes, including 97 NBS-LRR genes in the current version of the diploid *V*. *corymbosum* genome sequence. This represents about ~0.4% of all the predicted ORFs in this species, while the closely related cranberry genome contains 63 NBS genes (61 nTNLs and 2TNLs), which represents ~0.17% of the total predicted ORFs^37^. Thus, both the number and the relative representation of NBS-LRR genes is clearly higher in blueberry than in the cranberry genome.

Although the blueberry genome assembly used here is only a draft assembly, it is unlikely that the number of NBS genes has been dramatically underestimated. The same version of the genome has already been used successfully to enable formation of high-quality gene models similar in structure and number to those of many other well-annotated sequencing projects^32^. Therefore, we anticipate that the final number of NBS-LRR genes will only change slightly as new genome versions become available.

The accuracy of genome annotations is another factor that may be affected as new versions become released. However, several of our results act as independent lines of evidence indicating that the identified NBS-encoding genes may be functionally involved in plant defense. The first line of evidence comes from the high homology with previously described NBS-LRR genes from other species. This allowed us to explore the relationships between blueberry and other species by comparing our predicted proteins with NBS-LRR proteins hosted in the RefSeq database. The two species that showed highest identity with blueberry NBS proteins (apple and grape), however, are members of the subclass rosids and not the asterids, where blueberry belongs. The reason for this finding has to do with the low number of sequences present in the RefSeq database from the asterids species. For instance, two Ericaceae that are closely-related to blueberry, *V*. *macrocarpon* and *R*. *formosanum*, account for only 108 and 0 proteins in the RefSeq database, while the distantly related asterids, *I*. *nil* and *D*. *carota*, are represented by 51,054 and 44,660 proteins in the database, respectively (accessed on July 4, 2017). A second reason is the objective similarity between blueberry and grape^38^. Although *V*. *vinifera* has fewer sequences in the database (41,220) than apple (60,648), it shows more hits with the blueberry proteins. Both are woody species, berry-producing plants, with long generation times. Thus, they must share some morphological and biochemical characteristics. These physical and metabolic similarities may result in the sequence similarities between the species, despite blueberry and grape being members of different taxonomic classes^32,39,40^.

A second line of evidence for the role of the NBS genes in defense mechanisms is a clear enrichment of CREs in the promoter of the NBS sequences. *Cis*-acting elements are specific binding sites for proteins involved in the initiation and regulation of transcription. Studies on contribution of CRE provide insight into the promoter activity and improve our basic understanding of gene regulation^41,42^. We expected that promoters of functional NBS genes would contain a higher number of known CREs involved in defense and plant pathogen-specific responses. We tested 2,000 control sets to obtain a robust statistic of the motifs that were overrepresented in the real biological sequences. The equivalent control regions helped not only to identify motifs that are over-represented in the real biological sequences but also to show that the enriched CREs are more common in specific regions. The blueberry promoters showed a positional bias toward the 900–800 nucleotides upstream from the TSS, whereas the control set produced CREs with a uniform distribution in the promoter region. The difference in distributions is consistent with the view that the blueberry sequences are meaningful regulatory sequences, which according to their biological function contain multiple CREs to which transcription factors can bind, while the control set comprises just randomly occurring elements that are expected not to follow any particular pattern.

A third line of evidence comes from the examination of the GO terms ascribed to the NBS proteins. We used the suite Blast2GO to assign functional annotations to the predicted NBS proteins. There is a strong bias toward molecular function GO terms described as “…binding”. Analyzing biological process GO terms, the “defense response” theme arises. To improve accessibility and provide a resource that may facilitate the analysis to other researchers, the annotation files in Blast2GO format can be accessed via the publicly accessible Blueberry repository (https://github.com/jdieramon/BlueberryProject). Additionally, comparing gene ontology annotations of a given dataset to that of a well-studied transcriptome offers general information on the distributions of the functional categories. As expected, the gene models presented in this study show an overabundance of the terms ‘ADP binding’, ‘signal transduction’ and ‘defense response’. In summary, GO term analysis is consistent with the involvement of these genes in recognition of conserved binding sites and induction of defense responses.

### Recent duplication in blueberry NBS-LRR genes

Examination of the proportion of multi-gene families across similarity/coverage thresholds can be used to distinguish recent duplications of NBS genes in plant genomes. Blueberry NBS genes were mainly (over 50%) generated by recent duplications, according to standard criteria (coverage >70% and identity >70%), and more than 35% of NBS genes were still grouped the same based on strict standards (>80%). In line with recent findings in other woody species, this suggests that recent duplications have likely dominated the expansion of NBS genes in the blueberry genome. The Ks value is commonly used as a molecular clock to measure the time elapsed since gene duplication^43^. The frequency distributions of individual Ks values peak at 0.25–0.35 (52.2% duplications). Therefore, the Ks distribution also supports the hypothesis that recent duplication events resulted in the expansion of NBS genes.

As expected when we use the stringent criteria (>80%), we detect fewer multi-gene families, however, the stringent criterion does not represent a significant change in the average number of blueberry genes in a family. These results suggest that the expansion of the number of NBS-encoding genes was mainly caused by an increase in divergent gene groups and not by an increase in the number of genes within a group. The term “expansion of diversity” has been coined by Zhou *et al*.^44^ to describe the mechanism of expansion of diversified NBS-LRR genes. A plausible explanation is that plants benefit from diversifying NBS genes because they can then recognize more gene products from pathogens, thus increasing their ability to induce defense responses. To test this hypothesis, those authors proposed to investigate the similarity among closely related gene pairs (<5% divergence). In this study, two NBS genes on scaffold00001 and scaffold00509 (<1% divergence) make up 1.9% of the NBS genes (or 2.1% of the NBS-LRR genes) in the blueberry genome, roughly similar to the value for the rice (2.3%) or *Arabidopsis* (2.7%) genomes^44^. Therefore, no difference is observed in the number of almost identical genes in the blueberry genome. This seems to support the idea that the expansion of the NBS genes in blueberry is caused by the diversifying mechanism.

### Different evolutionary patterns of nTNL and TNL genes

Previous studies have interpreted the diverse evolutionary patterns in NBS-encoding genes as the result of adaptation, which allows plants to cope with specific ecological environments^25,45^. From various perspectives, our data here, along with previous studies, support that TNLs and nTNL genes exhibit different evolutionary patterns. Firstly, independent evolution of the TNLs and nTNLs could be detected in the phylogenetic tree by clear division into two parts. This division into two groups of NBS genes is well known and has been established for over a decade. Nevertheless, recent studies support the idea that NBS genes are actually composed of three anciently diverged classes named TNL, nTNL and RNL^46–48^. In blueberry, we have found one single copy of an RNL gene. Until recently, RNLs have been viewed as part of the nTNL class. In our study, the RNL sequence classified into the nTNL class. It was, however, not embedded into, but sister to one of the nTNL subclades. It is remarkable that we found a single copy of an RNL, because neither of the two plant species closely-related to blueberry, *Rhododendron* spp and *V*. *macrocarpon*, for which NBS genes have been analyzed, identified any RNL sequences^37,49^. This suggests that RNLs may not have expanded in the Ericaceae family. In addition, according to the phylogenetic tree, the branch length of TNL clade is significantly longer than that of nTNLs, suggesting than TNLs might have evolved faster than non-TNLs^24,25,45,47^. More support for different evolutionary patterns is that nTNL genes far outnumber TNLs, both in terms of number of gene families and proportion of multi-genes. This may be caused by a lack of TNL duplications and/or by a higher TNL loss rate^47,48,50^. Finally, NBS classes show distinct duplication history. As stated above, NBS genes appear to have been mainly generated by recent duplications. However, even though more than 35% of NBS genes are still grouped the same based on strict standards of 80%- *vs*. 70%-criteria, none of the TNL multi-gene families remained grouped under the strict 80%-criteria. Thus, the TNL class is less diversified, and the nTNLs have undergone more recent duplications than TNLs. All these discrepancies support the idea of different evolutionary patterns for TNL and nTNL genes.

### Concluding remarks

Having tags for the most important resistance alleles would aid greatly in the identification of elite, resistant germplasm and make it easier to pyramid multiple resistances into single cultivars. Based on a genome-scale survey, we identified 106 NBS-encoding genes in blueberry. We found that recent duplications generated the higher proportion of multi-genes. Several data support that the expansion of NBS genes in blueberry is caused by an expansion of diversity. First, we estimated that gene duplication is mainly caused by an increase in divergent gene groups and not by an increase in the number of genes within a group. Subsequenty, we found different evolutionary patterns of TNL genes and nTNL genes. The differential evolutionary patterns likely reflect an adaptative strategy against a specific array of co-evolving blueberry pathogens. We hope our data will help broaden insight into disease resistance traits in the species. Our study also provides a foundation for further comparative genomic analyses and a framework to trace the dynamic evolution of NBS genes on a large time-scale within the Ericaceae family. As more genomic data and better genome assemblies become available in the Ericaceae, NBS-LRR genes should be further studied among phylogenetically related species to gain a better understanding of their long-term evolutionary histories.

## Methods

### Retrieval and identification of blueberry NBS-encoding *R* genes

Assembly and annotation of the blueberry genome v1 was downloaded from the Genome Database for Vaccinium (GDV, https://www.vaccinium.org). The complete set of NBS-encoding genes was identified in the draft genome of blueberry using a reiterative process. First, a set of NBS protein coding sequences from *Vitis vinifera*^24^ was used as query against the blueberry database using a TBLASTN search and a threshold of *E* = 1e-5. Only the top hit was considered and predicted proteins from the blueberry genome were scanned using the Hidden Markov Model (HMM) corresponding to the Pfam NBS (the central NBS domain is also known as NB-ARC) family (Pfam: PF00931; http://pfam.xfam.org^51^). Sequences of less than 50% of the full-length NB-ARC domain were excluded from further analysis. In a second step, sixty-one possible candidates were expanded to 2,000–3,000 bp from both ends and the expanded nucleotide fragments were annotated using the gene-finding programs FGENESH (http://www.softberry.com) and GENSCAN (http://genes.mit.edu/GENSCAN.html) to obtain information on complete ORFs and on intergenic regions. Finally, each of these sequences was surveyed to determine whether they encoded TIR, CC, or LRR motifs using the Pfam database (http://pfam.xfam.org/), SMART protein motif analysis, (http://smart.embl-heidelberg.de/^52^, and COILS program to detect CC domains with a threshold = 0.9 (http://toolkit.tuebingen.mpg.de/pcoils;^53^. Detailed information obtained on protein motifs, domains and families was used to classify these NBS-encoding genes. Proteins with no match to NBS proteins (BLASTP search against the nr database at NCBI) were excluded from further analysis and the candidate list was eventually narrowed to 35 annotated NBS genes. The full-length NB-ARC domains of these sequences were then used as a query to search the entire blueberry genome to identify additional proteins through iterations over the blast-, extension-, and annotation- steps until we could not retrieve any more new sequences. After alignment, all identical sequences were checked manually and overlapping sequences were discarded. The final list was comprised of 106 NBS genes.

### Gene structure, prediction of conserved motifs and gene duplications

To investigate the diversity and structure of NBS-encoding genes, the exon/intron structures were obtained using the online Gene Structure Display Server (http://gsds.cbi.pku.edu.cn/) with both coding sequences and genomic sequences^54^. The predicted amino acid sequences of members of the TNL and nTNL subfamilies were subjected to motif analysis using the Multiple Expectation Maximization for Motif elicitation (MEME) system (http://meme.nbcr.net/meme/) under the following conditions: (1) optimum motif width was set to 15 and 20; (2) maximum number of motifs was designed to identify a number of motifs with an *E*-value < 1e-10. NBS gene duplication events were also investigated. To identify duplicated gene pairs, we defined a gene family according to the following criteria: (1) the alignable nucleotide sequence covered >70% of the longer aligned gene, and (2) the nucleotide identity between the sequences was at least 70%^24,55^.

### Anchor NBS-encoding genes to the diploid blueberry genetic linkage map

In order to anchor SSR markers from the most recent diploid blueberry genetic linkage map to blueberry draft scaffolds^56^, *in silico* PCR (http://hgwdev.cse.ucsc.edu/~kent/src/) was conducted with the following parameters: tileSize = 11, stepSize = 5, minPerfect = 15, minGood = 15. All SSR marker primers were adopted from Schlautman *et al*.^56^.

### Sequence alignment and phylogenetic analysis

For phylogenetic analysis, the alignments of amino acid sequences from nTNL and TNL genes based on conserved NBS domains (Ploop to MHDV) were performed using MUSCLE program as implemented in the Molecular Evolutionary Genetics Analysis software-MEGA ver. 5.1.^57^ with default options and manual corrections. Genes with short NBS domains (lacking more than 1 motif) were eliminated from the matrix because these interfered with a fine alignment and the subsequent construction of phylogenetic trees^47,48^. After removing short sequences with deletions, 94 NBS sequences were finally used to reconstruct NBS gene phylogeny for blueberry. The phylogenetic tree was constructed by using the maximum likelihood method. The reliability of the interior nodes was assessed using 1000 bootstrap replicates. The resulting alignments were then used to guide the alignments of the nucleotide coding sequences. The synonymous nucleotide substitutions Ks among triplets encoding the amino acids of NBS-encoding genes was calculated for each class for each of the groups determined by the phylogenetic tree with the MEGA program.

### Functional annotation

The Gene Ontology Functional Annotation Tool Blast2GO version 3.3.5^58,59^ was used to assign GO identities and enzyme commission numbers to the predicted NBS blueberry proteins. For the annotation, the following configuration settings were used: BLASTP against NCBI non-redundant (nr) protein database, *E*-value filter ≤ 10^−6^, length cutoff of 33, maximum 10 BLAST hits per sequence, and annotation cutoff of 50. Furthermore, to improve annotation, InterProScan was performed and results were merged to GO annotation. Blast2GO also enabled analysis related to over-representation of functional categories through the Gossip package^60^ for statistical assessment of annotation differences between two sets of sequences, using Fisher’s exact test for each GO term. False discovery rate (FDR < 0.01) was used to adjust for multiple testing.

### Identification and analysis of the promoter regions

For each predicted NBS-encoding gene we aimed to obtain the promoter sequence (~1500 bp from the ATG start codon) based on the information on the location of the genes assigned to scaffolds. Analyses on occurrence and distribution of *cis*-regulatory elements (CREs) over a given promoter were performed using standard Python scripts. The expected frequency of each motif was calculated using the average G + C content of 37% observed in the blueberry dataset. Probabilities were estimated based on control sets (2,000 Monte Carlo simulations, each set n = 65 with 1500 bp length). The characterized CREs included the three most common CREs found in the promoter sequences of the NBS-encoding genes in chestnut (DOFCOREZM [AAAG], CACTFTPPCA1 [YACT], and CAATBOX 1 [CCAAT]^61^; three CREs found in defensin promoters (GT1GMSCAM4 [GAAAAA], RAV1AAT [CAACA], and motif CTCTT^41^); three element regulators of abscisic acid responsiveness, dehydration and low temperature (ABRE [ACGTGTC], ATHB5 [CAATNATTG] and MYCATERD1 [CATGTG]^62–64^); and a binding site for the transcription factor MYC2 (G-box [CACATG]^65^).

### Data availability

The datasets generated during the current study are available in our Blueberry repository (https://github.com/jdieramon/BlueberryProject).

## Electronic supplementary material

Supplementary Information
