# Supplementary material for: Genome-scale examination of NBS-encoding genes in blueberry
Source: Sci Rep. 2018 Feb 21;8:3429. doi: 10.1038/s41598-018-21738-7 (PMC5821885; doi:10.1038/s41598-018-21738-7)
Supplement: Supplementary file 1 — Supplementary Information [file 41598_2018_21738_MOESM1_ESM.pdf]

Genome-scale examination of NBS-encoding genes in blueberry

Jose V. Die, Belen Roman del Castillo, Xinpeng Qi, Lisa J. Rowland

Supplementary Information

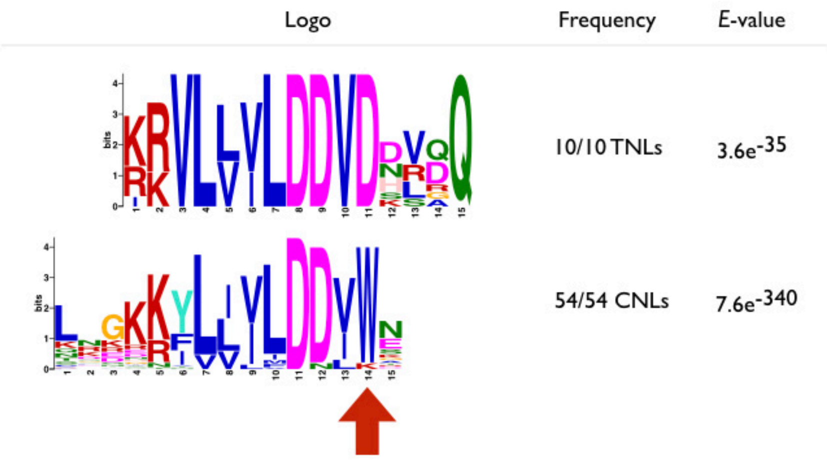

Supplementary Figure S1. **Analysis of the kinase-2 in the NBS domain.** The 'D' residue is shown in all the TNL proteins, whereas the 'W' residue is predominant in the CNL proteins. Sequence logo representation was generated from multiple alignments with MEME software.

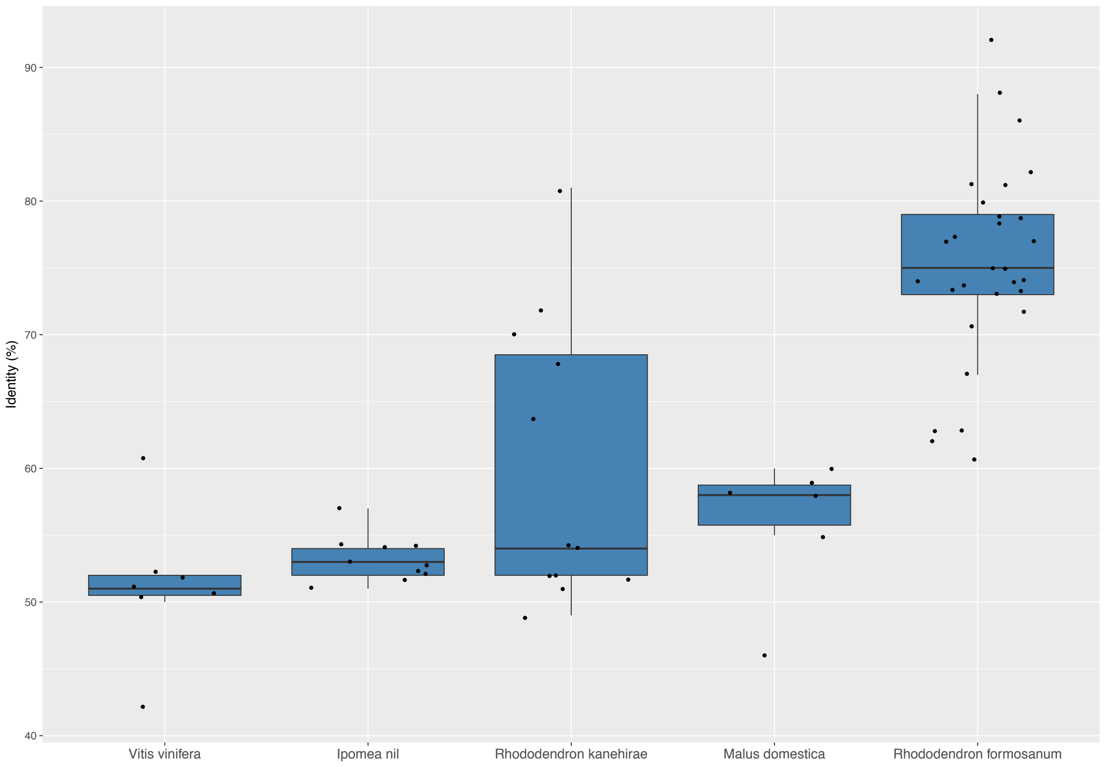

Supplementary Figure S2. Comparative analysis between blueberry NBS sequences and other species. Boxplot of identity distribution scores by protein nr database. Blueberry NBS sequences were used as queries against the nr database and the identity from the best-matching NBS protein for each blueberry sequence was recorded. Figure shows the five species with higher number of hits.

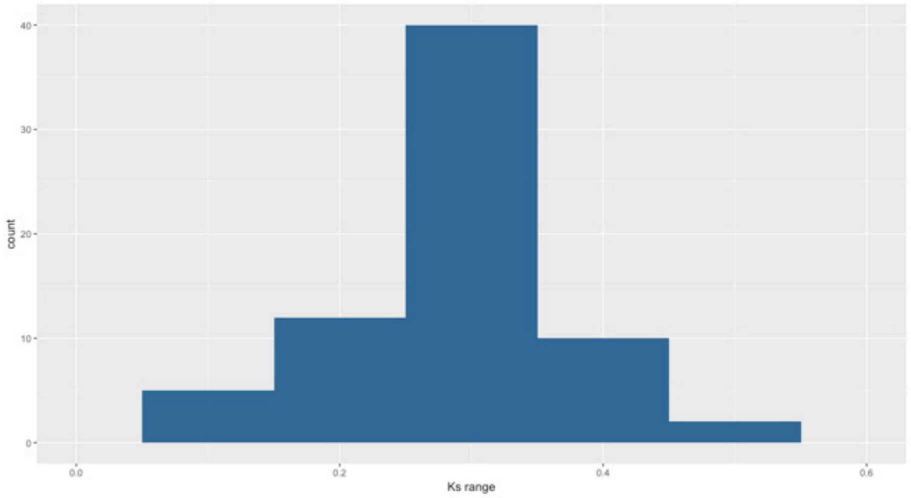

Supplementary Figure S3. Distribution of Ks values in blueberry.

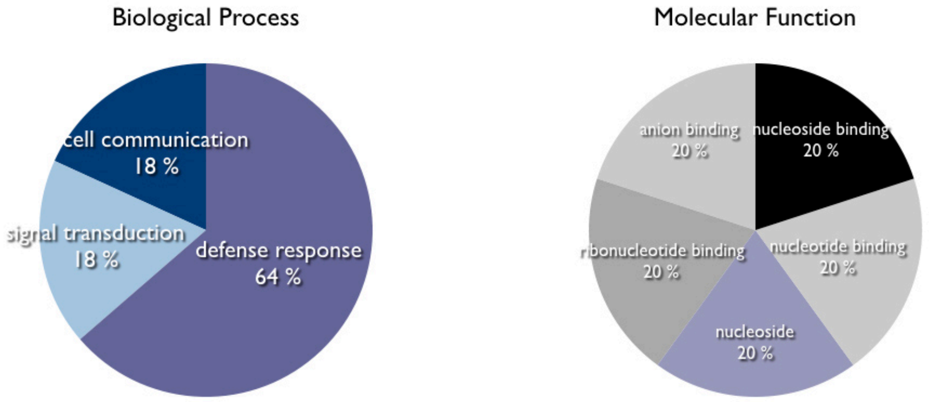

Supplementary Figure S4. Functional classification of Gene Ontology terms regarding 'biological process' and 'molecular function' according to blast2GO (level 4). Only terms represented by more than 10% of the sequences in any of the groups are shown.

Supplementary Table S1: Significant enrichment of GO terms for the blueberry NBS set related to several blueberry transcriptomes. Enrichment was measured by Fisher's exact test followed by multiple testing corrections. F= molecular function; P= biological process.

106 NBS sequences compared with 1,460 sequences regulated in flower buds exposed to 0 chill units [1]

**GO ID**	**GO term**	**Ontology**	**FDR**	***P*-value**
GO:0006952	ADP binding	F	7.1E-150	2.2E-153
GO:0006952	defense response	P	2.40E-17	1.5E-17

106 NBS sequences compared with 1,536 sequences regulated in flower buds exposed to 400 chill units [1]

**GO ID**	**GO term**	**Ontology**	**FDR**	***P*-value**
GO:0043531	ADP binding	**F**	1,2E-151	3,8E-155
GO:0006952	defense response	**P**	2,6E-20	1,6E-22

106 NBS sequences compared with 18,143 sequences regulated during fruit development [2]

**GO ID**	**GO term**	**Ontology**	**FDR**	***P*-value**
GO:0043531	ADP binding	**F**	1,7E-194	5,5E-198
GO:0006952	defense response	**P**	1,8E-51	1,0E-53
GO:0007165	signal transduction	**P**	6,6E-05	5,5E-07

[1] Die JV, Rowland LJ. Elucidating cold acclimation pathway in blueberry by transcriptome profiling. Environmental and Experimental Botany. 2014;106:87–98.

[2] Gupta V, Estrada AD, Blakley I, Reid R, Patel K, Meyer MD, et al. RNA-Seq analysis and annotation of a draft blueberry genome assembly identifies candidate genes involved in fruit ripening, biosynthesis of bioactive compounds, and stage-specific alternative splicing. GigaScience 2015 4:1.
